# The Impact of Transcutaneous Electrical Nerve Stimulation (TENS) on Acute Pain and Other Postoperative Outcomes: A Systematic Review with Meta-Analysis

**DOI:** 10.3390/jcm13020427

**Published:** 2024-01-12

**Authors:** Dmitriy Viderman, Fatima Nabidollayeva, Mina Aubakirova, Nurzhamal Sadir, Karina Tapinova, Ramil Tankacheyev, Yerkin G. Abdildin

**Affiliations:** 1 Department of Surgery, School of Medicine, Nazarbayev University, Astana 010000, Kazakhstan; mina.aubakirova@nu.edu.kz (M.A.); nurzhamal.sadir@nu.edu.kz (N.S.); karina.tapinova@nu.edu.kz (K.T.); 2 Department of Anesthesiology, Intensive Care and Pain Medicine, National Research Oncology Center, Astana 010000, Kazakhstan; 3 Department of Mechanical and Aerospace Engineering, School of Engineering and Digital Sciences, Nazarbayev University, Astana 010000, Kazakhstan; fatima.nabidollayeva@nu.edu.kz (F.N.); yerkin.abdildin@nu.edu.kz (Y.G.A.); 4 Department of Minimally Invasive Surgery, National Research Neurosurgery Center, Astana 010000, Kazakhstan; ramiltankacheyev@gmail.com

**Keywords:** acute pain, postoperative pain, transcutaneous electrical nerve stimulation, adverse events, hospital stay

## Abstract

This study aimed to investigate the efficacy and safety of transcutaneous electrical nerve stimulation (TENS) in postoperative acute pain control. PubMed, Scopus, and Cochrane Library were searched on 1–8 December 2022, for randomized controlled trials on the analgesic effects of TENS. The outcomes were pain intensity and opioid use (primary), and postoperative (PO) adverse events, blood pressure, and the duration of hospital stay (secondary); PROSPERO CRD42022333335. A total of 40 articles were included in the meta-analysis. Pain intensity at rest and during coughing for all types of surgeries combined was lower in the TENS group (standardized mean difference (SMD) = −0.51 [−0.61, −0.41], *p* < 0.00001, 29 studies, and −1.28 [−2.46, −0.09], *p*-value = 0.03, six studies, respectively). There was a statistically significant decrease in morphine requirements, as well as in the incidence of postoperative nausea and vomiting, dizziness, and pruritus. There was no difference between the groups in postoperative pain intensity during walking, in blood pressure, and only a borderline difference in the length of hospital stay. The subgroup analysis by surgery type did not show significant differences between the groups in pain severity at rest. Thus, TENS has a potential for pain control and postoperative recovery outcomes.

## 1. Introduction

Acute pain is the most common symptom and patient complaint during the acute postoperative period. It can contribute to numerous unwanted physiological and pathological effects, such as cardiovascular activation, resulting in tachycardia, elevated blood pressure, and increased myocardial oxygen demand; it can also impair respiratory, endocrine, and other system impairments. For patients undergoing simple surgical procedures, pain is one of the most frequent reasons for overnight hospital stays [1,2]. Postoperative pain is also a major cause of prolonged hospitalization, leading to a potential increase in morbidity after surgery [1]. Improved pain management may enhance postoperative recovery and reduce morbidity [3].

Prescribing opioids for the management of moderate to severe pain often results in side effects, with the most common being nausea, vomiting, intestinal hypomotility, and respiratory depression. Interventional modalities of acute pain management, such as epidural analgesia, some newer plane blocks, including transversus abdominis plane block, erector spinae plane block, and quadratus lumborum block, can provide effective pain management but require experienced specialists and can result in complications, such as local anesthetic systemic toxicity [4,5,6,7,8,9,10,11]. Multimodal analgesia is usually recommended for pain reduction [3,12]. This approach may include opioids, regional analgesia, low-dose ketamine, and neurocognitive modalities [12]. Nonpharmacologic methods, including transcutaneous acupoint electrical stimulation (TENS) and “transcutaneous acupoint electrical stimulation” (TAES), have been studied to improve postoperative pain control and reduce analgesic drug requirements [13,14,15].

TENS is a physical method of controlled low-voltage electrical nerve stimulation used for the reduction of pain. The electricity is conducted by electrodes placed on the skin [16]. TENS is a non-invasive, portable, compact, easy-to-use, and safe method of pain control [16]. In the postoperative period, TENS is used as an add-on pain management modality to standard analgesics rather than a stand-alone modality. The sterile electrodes are applied parallel to the surgical incision, and additional electrodes are placed over the thoracic spinal nerves in the corresponding area [17]. The possible advantages of TENS in postoperative pain management include faster mobilization and improvement in deep respiratory function and coughing, which might also shorten the time to discharge from the hospital [17].

The original theory suggested that the activation of descending inhibitory pathways might be the mechanism of action of TENS. Previous studies also supported the mechanism of segmentally mediated inhibition. Therefore, TENS appears to activate both descending and segmental inhibition [18]. The analgesic effect of TENS is also produced by the pain gate control theory, which is characterized by an attenuation of the nociceptive stimulation of afferent fibers of large diameter in the dorsal horn [19]. Conventional TENS activates the A-α and A-β fibers, alleviating the pain. TENS also activates the endogenous opioid system. This activation is produced by high and low-frequency stimulations [20].

Two decades ago, a meta-analysis comprising 21 studies examined the use of TENS in various surgeries and its effect on opioid consumption [21]. However, more studies have been published over the past twenty years; therefore, there is a need for additional analysis of the currently available data. Recently, meta-analyses have been conducted on the analgesic effects of TENS in orthopedic [22], pulmonary [23], inguinal hernia repair [24], gynecological [25], and cardiothoracic surgical interventions [26]. A recent large meta-analysis of 381 studies, comprising almost 25,000 patients, studied the analgesic effect of TENS in all-cause pain [27]. The authors state that aggregating pain intensity data regardless of the underlying medical condition is appropriate, as there is a lack of conclusive evidence establishing a connection between TENS outcomes and factors such as pathology, pain characteristics, medical diagnoses, or clinical context [27]. While this study comprises all types of pain, including chronic and non-surgical, it is important to also examine the effect of TENS on acute postoperative pain.

While these previous publications have examined the effect of TENS on postoperative pain, the evidence regarding the impact of TENS on pain and other postoperative outcomes is still inconclusive. Therefore, the goal of this work was to assess the efficacy and safety of TENS in acute postoperative pain management, as well as its influence on opioid consumption, ICU and hospital stay, and the rate of postoperative complication. We hypothesize that the use of TENS in various surgeries lowers pain scores and opioid consumption and might be associated with a reduction in postoperative adverse events and hospital length of stay.

## 2. Materials and Methods

### 2.1. Protocol

We used the PRISMA guidelines [28] and the Cochrane Handbook for Systematic Reviews of Interventions [29]. The protocol was registered in the PROSPERO database (CRD42022333335). We searched for suitable articles in PubMed, Scopus, and the Cochrane Library, published before December 2022. No gray literature was searched. We used the following search terms and combinations: (“transcutaneous electric nerve stimulation” OR (“transcutaneous” AND “electric” AND “nerve” AND “stimulation”) OR “transcutaneous electric nerve stimulation” OR (“transcutaneous” AND “electrical” AND “nerve” AND “stimulation”) OR “transcutaneous electrical nerve stimulation”) OR “transcutaneous acupoint electrical stimulation” AND (“pain, postoperative” OR (“pain” AND “postoperative”) OR “postoperative pain” OR (“postoperative” AND “pain”)). The filters used were “randomized controlled trials” and “English language”. No restrictions were used for the year or country of publication. Two authors independently worked on record screening and article searching. The search results retrieved from the mentioned databases were pooled in a spreadsheet, and duplicates were removed. Then, the two authors conducted the screening based on titles in accordance with the pre-specified inclusion criteria. The remaining articles were further screened based on the abstracts. Finally, the full texts were screened to identify those reporting the outcomes of interest. The final lists of articles were compared between the two authors. In case of disagreements, a third author was consulted to resolve the dispute.

### 2.2. Participants and Population

#### 2.2.1. Inclusion Criteria

Patients: postoperative patients with no limitations on age, gender, or type of surgery.

Intervention: use of TENS.

Comparison: sham or no TENS (with standard postoperative pain management).

Outcomes: pain and other clinically important postoperative outcomes (please see below).

Study: randomized controlled trials (RCTs).

#### 2.2.2. Exclusion Criteria

Types of studies: Study designs other than RCTs.

Outcomes: Studies not reporting the outcomes of interest.

### 2.3. Outcomes

The primary outcomes of our meta-analysis are postoperative (PO) pain (at rest, while walking, while coughing during a 72-h period postoperatively). The secondary outcomes were opioid consumption, postoperative adverse events, physiological parameters (blood pressure levels), and hospital stay duration (days).

### 2.4. Data Extraction and Statistical Methods

One author extracted and entered essential descriptive information (e.g., study goals and sample size). Another author extracted numeric data for meta-analysis from the studies. A third author further checked the descriptive and quantitative data, and disagreements were resolved by discussion. Data analysis was conducted using the software “Review Manager (RevMan) [Computer program]. Version 5.4. The Cochrane Collaboration, 2020”. The random-effects model was employed for the meta-analysis, in anticipation of heterogeneity resulting from the combination of various types of surgeries, patient populations, and the different scales used to report the outcomes of interest. All outcome variables were continuous. In some instances, we estimated statistics from the reported data using established statistical methods [30,31]. The effect size was reported as the standardized mean difference (SMD), mean difference (MD), or risk ratio (RR), with a 95% confidence interval. The utilization of SMD allowed for the standardized comparison of effect sizes across diverse outcome measures, such as pain scores and opioid use, accommodating the varied scales and units of measurement employed in the included studies. The risk ratio was used for analyzing dichotomous outcomes, such as adverse events, providing a measure of the relative risk between the TENS and control groups. Statistical significance was reported at *p* < 0.05. Heterogeneity was measured using the I^2^ statistic, which quantifies the proportion of total variation across studies due to heterogeneity. Additionally, the *p*-value of Cochran’s Q statistic was considered to evaluate the statistical significance of observed heterogeneity. A significance level of 0.1 was employed to determine whether the observed heterogeneity was statistically significant. Sensitivity analyses were performed for each outcome by running the model with the elimination of studies one by one and were reported only if the results were sensitive.

### 2.5. Assessment of the Methodological Quality and the Publication Bias

Two authors independently assessed the methodological quality. The Cochrane Risk of Bias tool 2.0 [32] was utilized to evaluate the methodological quality of the studies. The risk of bias was categorized as “high”, “low”, or “medium/some concerns”, based on the provided description of randomization and blinding procedures, as well as the reporting of results. Furthermore, we assessed the certainty of evidence using the Grading of Recommendations Assessment, Development, and Evaluation (GRADE) [33]. Five outcomes (pain at rest at 24 h, morphine consumption at 24 h, PONV, pruritus, and hospital length of stay) were evaluated for upgrading or downgrading based on the risk of bias, imprecision, inconsistency, and indirectness. Each of these outcomes received a certainty of evidence grading ranging from “very low” to “high”. Additionally, we conducted a comprehensive assessment of publication bias utilizing both funnel plots and Egger’s regression test.

## 3. Results

### 3.1. Article Search Results

**Figure 1:**
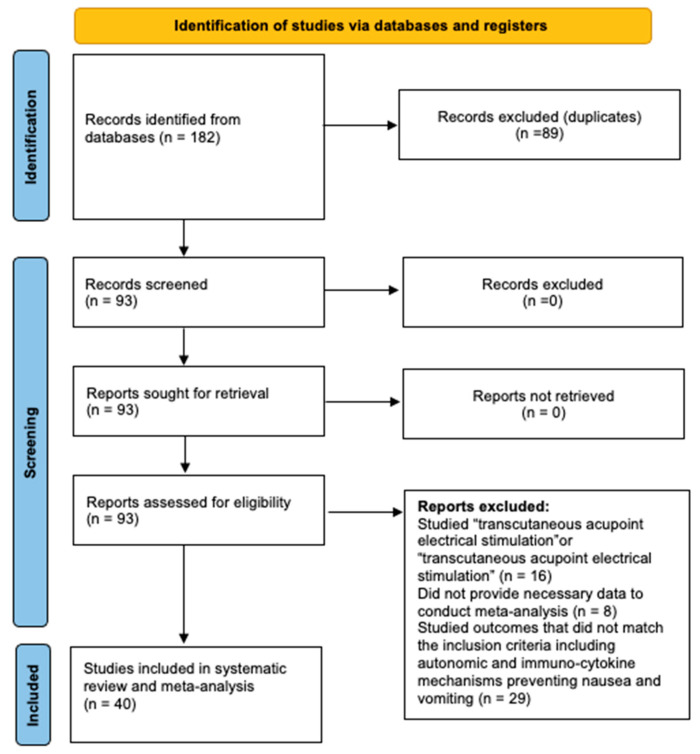
PRISMA diagram.

In total, 182 articles were initially identified (Figure 1). Of them, 89 duplicates were removed. Subsequently, 93 RCTs were screened. After screening the titles and abstracts, 53 articles were excluded. Finally, a total of 40 articles [2,13,34,35,36,37,38,39,40,41,42,43,44,45,46,47,48,49,50,51,52,53,54,55,56,57,58,59,60,61,62,63,64,65,66,67,68,69,70,71] with 2265 (TENS—1137, control—1128) patients were included in the meta-analysis (Table 1).

### 3.2. Assessment of Methodological Quality

Regarding methodological quality, 10 studies had a low risk of bias, while 29 studies were assessed as having “some concerns” regarding the risk of bias. One study had a high risk of bias. The detailed results of the quality analysis are presented in Table 2 [2,13,34,35,36,37,38,39,40,41,42,43,44,45,46,47,48,49,50,51,52,53,54,55,56,57,58,59,60,61,62,63,64,65,66,67,68,69,70,71].

### 3.3. Pain at Rest

**Figure 2:**
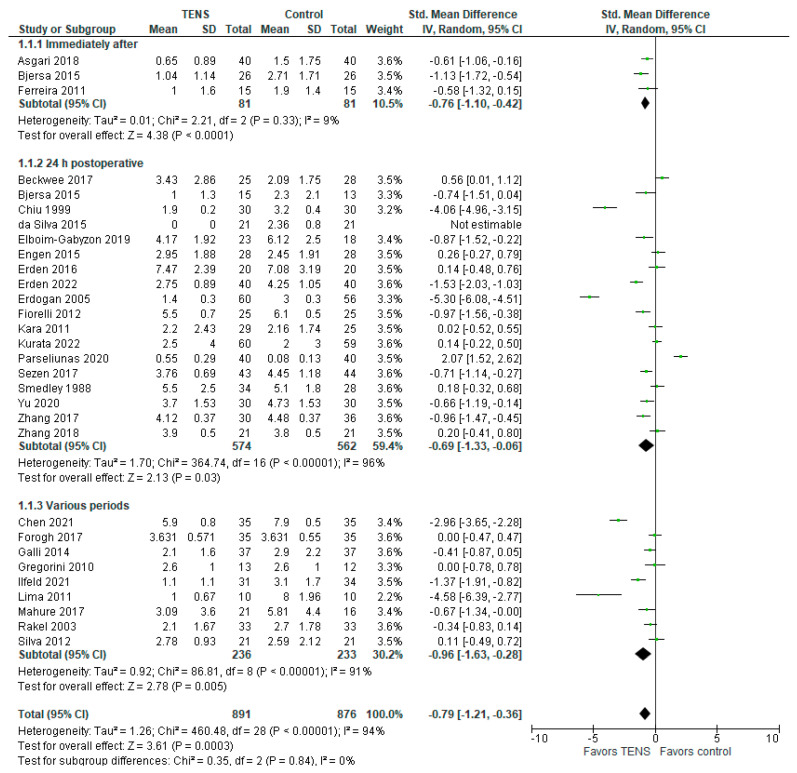
Pain at rest [2,13,34,35,36,37,38,39,40,41,42,44,45,47,48,49,50,51,52,53,54,55,56,57,58,59,60].

The forest plot in Figure 2 illustrates the pain intensity at rest measured immediately after surgery, 24 h post-surgery, and at various intervals. The overall model effect favors TENS over the control, indicating a standardized mean difference (SMD) on a 0-10 scale with a 95% CI of −0.79 [−1.21, −0.36], with a *p*-value less than 0.00001. However, it is important to note that the model shows substantial heterogeneity (I^2^ = 94%). The TENS group comprises 891 patients, while the control group consists of 876 patients. One study [72] was excluded from the meta-analysis due to the absence of a reported sample standard deviation.

Subgroup analysis further reinforces the superiority of TENS over the control across all three subgroups (‘immediately after surgery’, ’24 h after surgery’, and ‘various periods’), although with considerable heterogeneity for the latter two (I^2^ = 96% and I^2^ = 91%, respectively). In the ‘immediately after surgery’ subgroup, the SMD with a 95% CI is −0.76 [−1.10, −0.42], with a highly significant *p*-value < 0.0001, I^2^ = 9%. In the primary (24 h postoperative) subgroup, the SMD with a 95% CI is −0.69 [−1.33, −0.06], with a *p*-value of 0.03, I^2^ = 96%. The third subgroup, covering varied measurement times, such as ‘after TENS’ [13], ’12 h postoperative’ [59], ‘postoperative day 3’ [58], ‘postoperative day 7’ [34], ‘4 weeks postoperative’ [56], and instances with no provided information [39,55,57,60], shows a significant SMD with a 95% CI of −0.96 [−1.63, −0.28], with a *p*-value of 0.005, I^2^ = 91%. These results indicate statistically significant improvements in pain intensity for the TENS group in all the measured periods.

### 3.4. Pain at Rest for Specific Types of Surgeries 24 h PO

**Figure 3:**
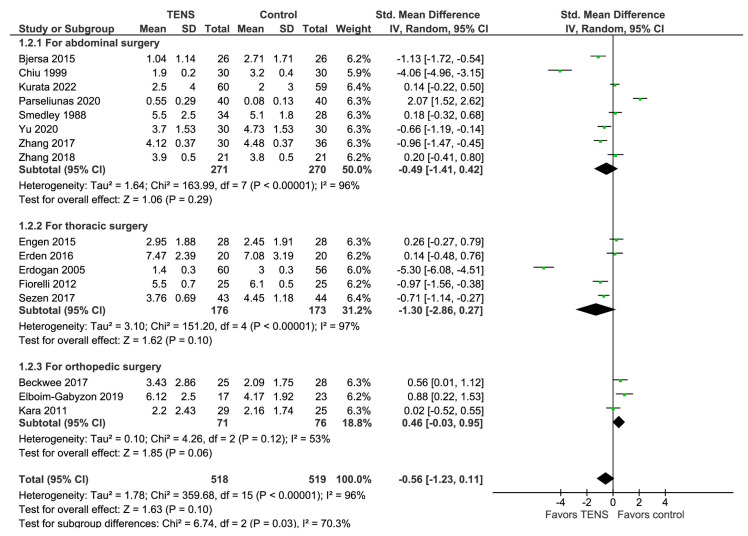
Pain at rest 24 h after different surgeries [2,36,38,41,42,43,44,46,47,48,49,50,51,52,53,54].

The pain intensity at rest, measured 24 h after three different types of surgeries (abdominal, thoracic, and orthopedic), is presented in the forest plot below (Figure 3). The overall effect of the model shows no significant difference between TENS and control (SMD on a 0–10 scale with a 95% CI = −0.56 [−1.23, 0.11], *p*-value = 0.10), and the model shows substantial heterogeneity with the value of I^2^ = 96%. However, the result is sensitive to the exclusion of the study by Parseliunas (2020), in which case the model favors the TENS group. The total number of patients in the TENS group is 518, and 519 in the control group. Two studies [35,45], representing the results for plastic and breast surgery, respectively, were excluded.

In terms of subgroup analysis, the model shows no significant difference between the TENS and control groups. The ‘For abdominal surgery’ subgroup yielded an SMD of −0.49 [−1.41, 0.42], *p*-value = 0.29; the ‘For thoracic surgery’ subgroup yielded an SMD of −1.30 [−2.86, 0.27], *p*-value = 0.10; and the “For orthopedic surgery” subgroup yielded an SMD of 0.46 [−0.03, 0.95], *p*-value = 0.06.

### 3.5. Pain while Walking (POD 1, POD 2)

**Figure 4:**
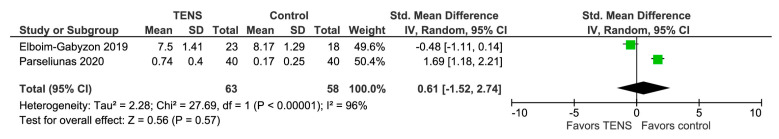
Pain while walking (POD 1, POD 2) [43,49].

The forest plot in Figure 4 illustrates the pain intensity while walking measured 24 h and 48 h after surgery. The overall effect of the model does not favor TENS over the control (SMD with a 95% CI: 0.61 [−1.52, 2.74], *p*-value = 0.57). This result is sensitive to the exclusion of the study by Elboim-Gabyzon et al., 2019 [43]. The model shows considerable heterogeneity (I^2^ = 96%), which is likely attributed to the limited number of included studies. Parseliunas et al., 2020 [49] reported pain while walking values for postoperative day 1 (POD 1), whereas Elboim-Gabyzon et al., 2019 [43] reported them for POD 2.

### 3.6. Pain at Coughing (POD 1, POD 3)

**Figure 5:**
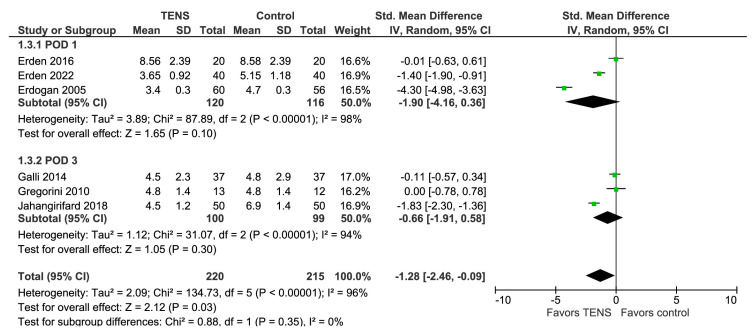
Pain at coughing (POD1, POD 3) [36,45,46,57,58,61].

The overall effect of the model favors the TENS group over the control group (SMD with a 95% CI: −1.28 [−2.46, −0.09], *p*-value = 0.03) (Figure 5). This result is statistically significant but sensitive to the exclusion of some studies [45,46,61]. Subgroup analysis reveals no significant difference between the groups. It is important to note that one study [57] did not provide the time of measurements, and as a result, we included it in the POD 3 subgroup.

### 3.7. Morphine Requirements (mg)

One study [66] reported ketorolac intake (mg), and we adjusted the values by multiplying them by 0.4, following the recommendation of the American Pain Society in 2003 and 2008 (https://cdn-links.lww.com/permalink/jpsn/a/jpsn_4_2_2015_04_23_manworren_jpsn-d-14-00050r2_sdc1.pdf (accessed on 15 December 2022)). Another study [38] reported IV oxycodone (mg) consumption, and we used a conversion factor of 1.5. Finally, one study [62] reported IV morphine consumption at 24 h; however, there was not enough information about the units (mL in the Table, but mg in the text), so we did not include this study in the analysis (the results were not sensitive to the values from this study).

Some studies [42,47,66] did not explicitly report the time of measurement, so we included them in the ‘No time info’ subgroup. The majority of the studies reported morphine requirements within 24 h after surgery (‘POD 1′ subgroup). One study [61] was excluded due to the absence of information about the sample standard deviation.

**Figure 6:**
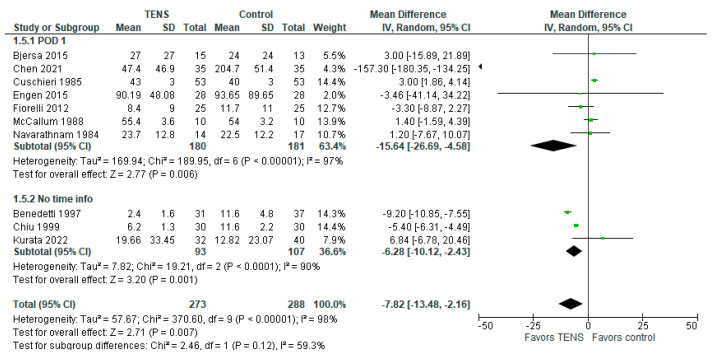
Postoperative morphine requirements (mg) [2,38,42,44,47,55,63,64,65,66].

The overall effect of the model favors TENS over control (MD with a 95% CI: −7.82 [−13.48, −2.16], *p*-value < 0.00001) (Figure 6). However, this result is sensitive to the exclusion of a study by Chen 2021 [55]. Broken down, on POD1, the use of TENS decreased morphine use by 15.64 mg [−26.69, −4.58], *p*-value = 0.006, I^2^ = 97%. In the “no time” subgroup, morphine use was decreased by 6.28 mg (−6.28 [−10.12, −2.43], *p*-value = 0.001, I^2^ = 90%) in the TENS group.

### 3.8. Postoperative Nausea and Vomiting

**Figure 7:**
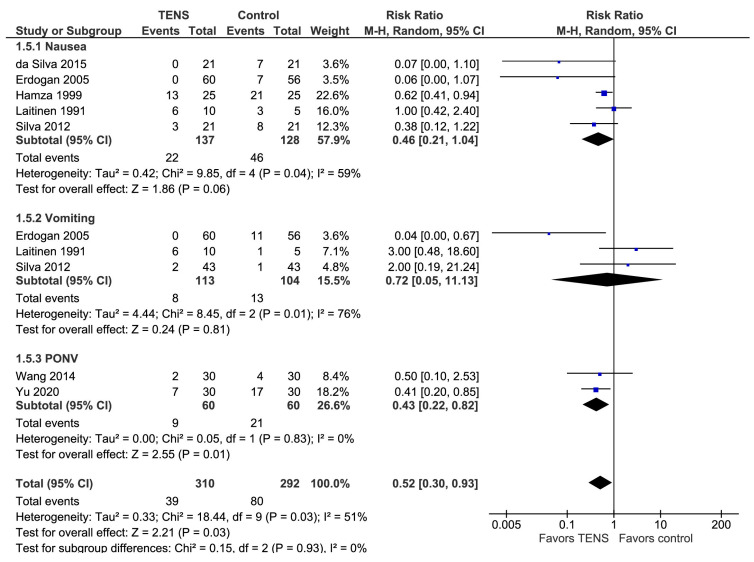
PO nausea and vomiting [13,35,46,52,67,68,71].

The overall effect of the model favors the TENS group over the control group (the risk ratio (RR) with a 95% CI: 0.52 [0.30, 0.93], *p*-value = 0.03; I^2^ = 51%) (Figure 7). It should be noted that the studies primarily reported the incidences of PONV on postoperative day 1 (POD 1) and postoperative day 2 (POD 2), but several studies did not explicitly report the time of measurement [13,35,46].

The subgroup analysis indicates that the model favors the TENS group over the control group in two subgroups, namely ‘nausea’ and ‘PONV’.

### 3.9. Other Adverse Events (Dizziness and Pruritus)

**Figure 8:**
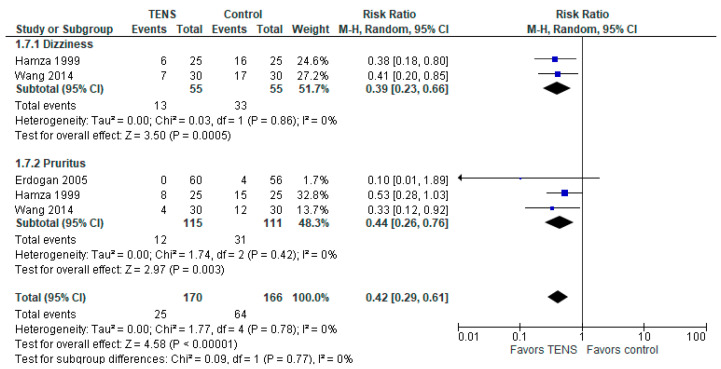
Other adverse events [46,67,71].

The overall effect of the model favors TENS over control (RR with a 95% CI: 0.42 [0.29, 0.61], *p*-value < 0.00001, I^2^ = 0%) (Figure 8). The model supports TENS over control in both subgroups: ‘dizziness’ and ‘pruritus’. Specifically, for dizziness, the RR for the TENS group is 0.39 [0.23, 0.66], *p*-value = 0.0005, I^2^ = 0%, 2 studies, 110 patients. For pruritus, the RR for the TENS group is 0.44 [0.26, 0.76], *p*-value = 0.003, I^2^ = 0%, 3 studies, 226 patients.

### 3.10. Hospital Stay Duration (Days)

**Figure 9:**
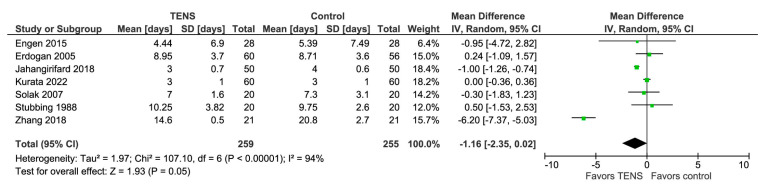
Hospital stay duration (days) [44,46,47,54,61,69,70].

The model shows no significant difference between TENS and control (MD with a 95% CI: −1.16 [−2.35, 0.02], *p*-value = 0.05; I^2^ = 94%) (Figure 9). The result is sensitive to the exclusion of any of these three studies: Erdogan 2005, Solak 2007, or Stubbing 1998, in which case, the model favors TENS.

### 3.11. Blood Pressure Postoperatively (mmHg)

**Figure 10:**
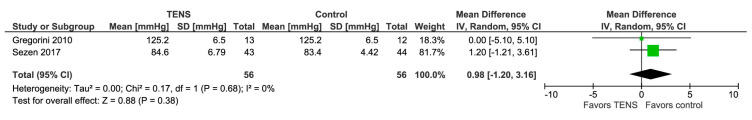
Blood pressure PO (mmHg) [58,50].

The model indicates no significant difference between TENS and control (MD with a 95% CI: 0.98 [−1.20, 3.16], *p*-value = 0.38; I^2^ = 0%) (Figure 10). Sezen et al., 2017 [50] reported the blood pressure values for postoperative day 1 (POD 1), and Gregorini et al., 2010 [58] reported these for postoperative day 3 (POD 3).

### 3.12. Publication Bias

Our findings from the analyses using the funnel plots and Egger’s regression test did not indicate substantial evidence of publication bias in the studies included in our meta-analysis regarding pain intensity.

**Figure 11:**
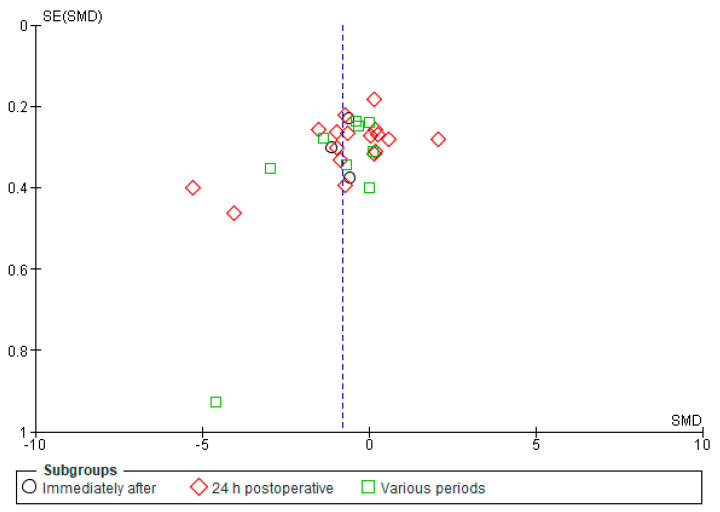
Funnel plot for pain at rest, the random effects model.

The funnel plot below for the pain intensity at rest (Figure 11) demonstrates a spread of study outcomes that resembles a slightly asymmetric distribution. This slight asymmetry could be attributed to the nature of the random effects model, accounting for potential heterogeneity among the included studies.

**Figure 12:**
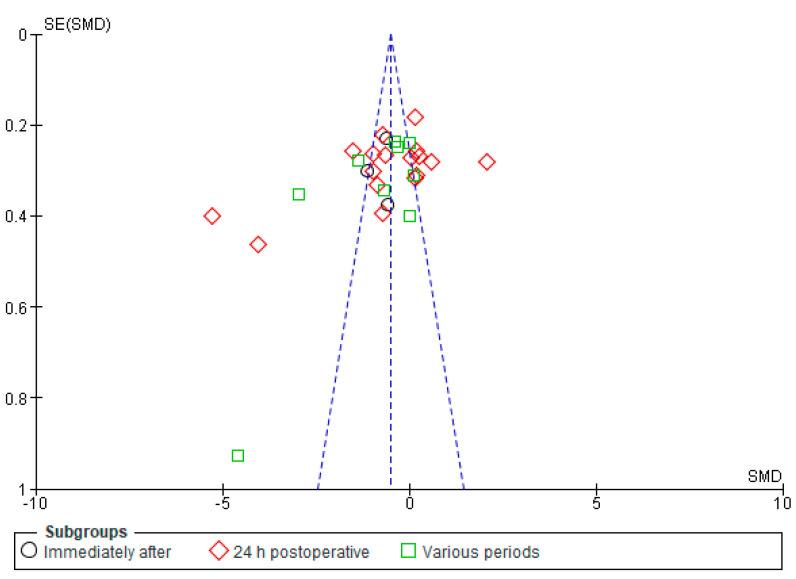
Funnel plot for pain at rest, the fixed effects model.

In contrast, the funnel plot under the fixed effect model (Figure 12) illustrates a more symmetric distribution of study outcomes. However, it is important to note that the fixed effect model assumes homogeneity across studies, which might not accurately represent the true variability seen in the data.

### 3.13. Certainty of Evidence

Table 3 provides the certainty of the evidence for five outcomes (pain at rest at 24 h, morphine consumption at 24 h, PONV, postoperative adverse events, and hospital length of stay). The certainty of evidence ranges from “very low” to “moderate”. The evidence profile (Table 4) contains information regarding the quality of evidence evaluation and the summary of findings for each of the studied outcomes. 

## 4. Discussion

Our meta-analysis revealed positive associations between TENS and various postoperative improvements, including reduced immediate and early postoperative pain, as well as diminished pain at coughing on days 1 and 3. Furthermore, TENS demonstrated effectiveness in decreasing morphine requirements, overall PONV, dizziness, pruritus, and, possibly, hospital length of stay. However, TENS did not show a significant impact on pain during walking. Similarly, sub-analysis based on the type of surgery did not reveal differences in pain scores.

A previously conducted large meta-analysis supports our findings regarding the pain-alleviating effect of TENS. The authors found that TENS reduced acute pain (surgical and non-surgical combined together) by −1.02 [−1.24, −0.79] on a ten-point scale, and postoperative pain by −0.92 [−1.15, −0.69] [27]. All studies combined (92 samples with 4841 participants reporting acute/chronic pain, (non)procedural, etc.) produced similar results: Pain scores in the TENS group were −0.96 [−1.14, −0.78] lower than in the placebo arm [27]. The researchers concluded that while TENS provided analgesic effects, the type of pain, the diagnosis, and the procedure did not affect the impact [27].

Unlike our study, meta-analyses concentrating on specific surgeries observed a statistically significant pain reduction in the TENS group compared to controls. A meta-analysis comprising 559 patients observed lower pain scores on POD one, two, and three in the TENS group compared to the placebo following inguinal hernia repair [24]. In a gynecological study, TENS was found to provide a pain-relieving effect comparable to that of opioids [25]. The use of TENS reduced pain scores at 12, 24, and 48 h following total knee arthroplasty (TKA), according to a meta-analysis of five studies comprising 472 patients [22]. Similarly, TENS reduced pain scores on the first five postoperative days following lung surgeries [23].

Regarding opioid consumption, a meta-analysis of 21 studies found that TENS reduced the postoperative use of opioids by more than 25% [21]. The TENS group consumed fewer opioids in the post-analgesia care unit than the opioid-only group following gynecological surgeries [25]. TENS reduced opioid use at 12, 24, and 48 h following TKA [22]. Similarly, lower pain scores at rest and on coughing were observed in the TENS group after cardiothoracic procedures [26].

Thus, while previous literature demonstrates the pain-relieving and opioid-sparing effects of TENS in the postoperative period following specific surgeries or generally for acute pain management, our study contributes insights to the existing literature on TENS by providing a comprehensive analysis of its effectiveness in postoperative pain management across various surgical contexts. This approach allows for a more generalized evaluation of TENS efficacy in postoperative pain control, offering valuable insights applicable to a wide array of clinical scenarios. Our study examines a comprehensive set of postoperative outcomes, including immediate and early postoperative pain, pain at different time points, pain during specific activities (coughing and walking), as well as morphine requirements, adverse events, and hospital length of stay. This holistic approach provides a more nuanced understanding of TENS’s impact on various aspects of postoperative recovery.

This meta-analysis observed a considerable heterogeneity in most outcomes. However, such heterogeneity was anticipated beforehand, given the differences in the durations of interventions, patient characteristics, control groups, surgical procedures, and outcome measures. Variations in TENS protocols, including differences in electrode placement, stimulation parameters, and treatment duration, introduced another source of heterogeneity. Although this heterogeneity posed a challenge in terms of combining and interpreting data, given that the effectiveness of TENS may vary across different contexts, a random effects model was used to account for the between-study variability. Furthermore, sub-group analysis was undertaken wherever possible to obtain more homogeneous results. Sensitivity analyses were also performed to help explore the sources of heterogeneity.

An important limitation of this research was the lack of extended follow-up information, which would have allowed for an assessment of the long-term advantages or potential complications associated with the use of TENS. Furthermore, the quality of the meta-analysis was contingent on the quality of the studies included. As evident from the Cochrane risk of bias, a number of studies had “some concerns” regarding the risk of bias, which subsequently affected the certainty of the evidence. Finally, challenges related to synthesizing data, such as differences in outcome measurement scales or reporting formats, complicated the aggregation of results and the conduct of a comprehensive analysis, as some studies had to be excluded due to these issues.

The implications of our meta-analysis extend both clinically and practically. For clinicians, our findings suggest that incorporating transcutaneous electrical nerve stimulation (TENS) into postoperative pain management protocols can offer tangible benefits, particularly in alleviating early postoperative pain and opioid requirements, and reducing adverse events. This information empowers healthcare providers to make informed decisions about the inclusion of TENS in multimodal analgesia strategies, enhancing overall patient care. Therefore, the study may serve as a guide for clinicians considering TENS as an adjunctive therapy in postoperative care.

From the research point of view, the study identified the need for investigations into the long-term effects of TENS and its impact on specific surgical contexts. Researchers could focus on conducting well-designed, prospective studies with extended follow-up periods to understand the sustained benefits and potential delayed adverse effects of TENS.

## 5. Conclusions

When considering all types of surgeries, the meta-analysis shows that TENS reduces pain intensity at rest (immediately after surgery and 24 h after surgery), pain intensity during coughing, morphine consumption, the incidence of PONV, and other adverse events, such as PONV, dizziness, and pruritus. We did not find a significant difference between the TENS group and the control group in reducing pain during walking. The subgroup analysis does not show significant differences between the TENS group and controls in pain severity at rest for thoracic, abdominal, or orthopedic surgeries.

## Figures and Tables

**Table 1:** Study characteristics. Abbreviations: RoM, range of motion; TKA, total knee arthroplasty; VAS, visual analog scale; LAS, linear analog scale; N, number; PONV, postoperative vomiting and nausea; TENS, transcutaneous electrical nerve stimulation; QoR, quality of recovery; NRS, numeric rating scale; TEAS, transcutaneous acupoint stimulation; QoL, quality of life [2,13,34,35,36,37,38,39,40,41,42,43,44,45,46,47,48,49,50,51,52,53,54,55,56,57,58,59,60,61,62,63,64,65,66,67,68,69,70,71].

Author, Year, Country	Study Goals	Age	N of Patients: Total (TENS/Control); % Male	% Male (TENS/Control)	Groups	Diagnosis	Comorbidities	Type of Surgery	The Timing of TENS	Method of Pain Measurement	Study Conclusions
Beckwee, 2017, Belgium [41]	Pain, knee RoM, analgesic use	71.8 (7.3) 72.9 (7.6)	53 (25/28)	32%/39.3%	TENS Sham	-	-	TKA	40 min	100 mm VAS before and after, daily	No effects on pain
Forogh, 2017, Iran [56]	Pain, IKDC, RoM	26 (4.1) 26.31 (4.33)	70 (35/35)	100%	TENS No TENS, both groups exercise	Injury to the ACL	-	Post-anterior cruciate ligament reconstruction	20 sessions, 4 weeks, 35 min/day	100 mm VAS	No effect on knee function and pain
Asgari, 2018, Iran [37]	Pain, fentanyl use, PONV	31.35 (4.89) 31.15 (6.28)	80 (40/40)	0%	TENS No TENS, 50 mg fentanyl	Ectopic pregnancy, infertility, ovarian cysts, ovarian torsion	-	Laparoscopic Gynecologic Surgery	20 min for patients who complained of pain	10-cm VAS before, and 5, 10, 20, 30 min after treatment	TENS is not superior to fentanyl for pain relief
Bjersa, 2014, Sweden [62]	Pain, QoR-40, extra analgesia use, EDA infusion rate, total TENS use time	69.1 65.5	20 (9/11)	56%/73%	TENS Sham	-	-	Pancreatic resection: Ad Modum Whipple pancreaticoduodenectomy	30 min sessions; for 24 h post-op	Pain-O-Meter, estimation on 100-mm scale	Supports use of high-frequency TENS
Bjersa, 2015, Sweden [38]	Pain, QoR-40, total analgesia use, time of TENS use	67.9 (11.6) 74.1 (10.3)	28 (15/13)	53%/86%	TENS Sham	Colon diseases and malignancies, unknown	-	Open colon resection	No time limits; each session—30 min; for 24 h post-op	Pain-O-Meter, estimation on 100-mm scale	Benefits of TENS
Cuschieri, 1985, UK [63]	Pain, morphine use, ABG	51 57	106 (53/53)	43%/40%	TENS Sham	-	-	Abdominal surgery	3 days post-op	LAS, before + after twice daily for 3 days	Results do not support TENS use
Galli, 2014, Brazil [57]	Pain	44.32 (9.98) 44.22 (8.21)	74 (37/37)	57%/38%	TENS Sham	Healthy kidney donors	HTN, asthma, gastritis, hypothyroidism	Open nephrectomy	For 1 h during first post-op day	NRS before and after	TENS decreases pain and increases max expiratory pressure
Hamza, 1999, USA [67]	PCA demands and doses, sedation, fatigue, discomfort, pain, nausea, side effects	43 (11) 44 (11) 45 (10) 43 (9)	100 (25/25/25/25)	0%	PCA + sham PCA + low-frequency TENS PCA + high-frequency TENS PCA + mixed-frequency TENS	-	-	Major gynecological procedures	Every 2 h during the day	100 mm VAS at baseline, 24, 48 h	TENS decreases post-op opioid analgesic use and opioid-related side effects
Laitinen, 1991, Finland [68]	Pain, BP, HR, RR, side effects	63.4 (7.8) 50.2 (8.6) 56.6 (11.5) 61.4 (8.4) 52.2 (8.4) 40.6 (11.4) 49.6 (16.9) 46.9 (14)	60 (10/10/20/20)	20%/0%/0%/0%/3%	Control Indomethacin Low-frequency TENS + indomethacin High-frequency TENS + indomethacin	Cholecystitis	-	Cholecystectomy	16 h	No/mild/moderate/severe every 4 h	Neither indomethacin nor TENS reduce the postoperative opiate requirement.
Rakel, 2003, USA [60]	Pain, walking function, vital capacity	20–77 40 (15)	33	48%	Pharmacologic analgesia + TENS Pharmacologic + sham TENS Pharmacologic only	-	End-stage renal disease, diabetes	-	15 min, 2–4 h between the sessions	NRS 0–20	Reduces pain and increases walking function post-op
Silva, 2012, Brazil [13]	Pain, PONV	52 (14) 44 (16)	42 (21/21)	7%	TENS Sham TENS	Cholecystitis	-	Laparoscopic cholecystectomy	30 min during 24h post-op	11-point VNS, VAS (0–10)	Decreases pain and PONV
Yu, 2020, China [52]	QoR, pain at rest, MMSE, PONV, medication use	48.5 (16.2) 45.9 (17.5)	60 (30/30)	0%	TEAS Sham TEAS	-	-	Gynecological laparoscopic surgery	30 min before anesthesia	100 mm VAS	Improves QoR, MMSE; reduces pain, PONV
Zhang, 2017, China [53]	Pain, bladder spasm episodes	64.5 (54–79)	66 (30/36)	100%	TENS No TENS	BPH, bladder disease	-	Bladder or prostate surgery	3 days post-op, each session for 60 min	VAS 0–10	Relieves post-op bladder spasms
Zhang, 2018, China [54]	Pain, time to first: defecation, flatulence, diet; LOS, HRV	68 (1.4) 64 (2.6)	42 (21/21)	86%/71%	TEAS Sham TEAS	GI cancers	-	Open abdominal surgery for cancers	1 h, twice daily, 3 d	VAS 0–10	Improves major post-op symptoms
Chiu, 1999, Taiwan [42]	Pain, total PCA morphine use, N of nurse calls for analgesia	53.1 (2.7) 56.0 (3.1)	60: (30/30)	75%	TENS on acupoints TENS on sham acupoints	Symptomatic hemorrhoids	-	Hemorrhoidectomy	Postoperative, 2 times a day	0–10	Complications—hemopericardium, better pain relief
Elboim-Gabyzon, 2019, Israel [43]	Pain, FAC, physical performance	78.06 (8.45), 80.26 (9.83)	41: (18/23)	13%/33%	TENS Sham TENS	Intertrochanteric or sub-trochanteric fracture	Yes	Hip fracture surgery	Postoperative	NRS 0–10	Pain relief
Benedetti, 1997, Italy [66]	Time to analgesia, total medication use, pain	-	103 106 112	Not given	TENS Sham TENS No TENS	Empyema, myasthenia gravis	-	Posterolateral thoracotomy, muscle-sparing thoracotomy, costotomy, sternotomy, and video-assisted thoracoscopy	1 h post-op, 1 h rest interval, 1h more	NRS 0–10	Useful for mild to moderate pain; ineffective for severe pain
Engen, 2015, USA [44]	Pain, analgesia use, patient satisfaction	61.5 (11.21), 61.8 (13.13)	56: (28/28)	30%/55%	TENS + opioids Opioids only	-	-	Thoracoscopic surgery	48 h post-operatively	VAS 0–10	No effect on pain or morphine use
Erden, 2016, Turkey [36]	Pain, analgesic use	54.9 (13.3), 50.0 (12.7)	40: (20/20)	70%/80%	TENS No TENS	Lung cancer	Chronic disease	Posterolateral thoracotomy	Postoperative 30 min	VAS	Reduces pain
Erdogan, 2005, Turkey [46]	Pain, FEV1, FVC, PaO2, PaCO2, doses of analgesia, sedation, side effects	55.6 (11.9) 52.93 (11.48)	116 (60/56)	63%/57%	TENS No TENS	Lung cancer	-	Posterolateral thoracotomy	For 20 min at 3-h intervals for 3 days	VAS 0–10	Routine use recommended
Ferreira, 2011, Brazil [40]	Pain	49 (14), 55.0 (14.9)	30: (15/15)	67%/53%	TENS Sham TENS	Lung cancer	-	Thoracotomy	Second post-op day	VAS 10 cm	Reduces pain severity
Fiorelli, 2011, Italy [2]	Cytokines, pain, respiratory function, medication usage	64 (1), 64 (4.1)	50: (25/25)	74%/61%	TENS Sham TENS	Lung cancer	-	Standard posterolateral thoracotomy	48 h post-op, 30 min	VAS 0–10	Reduces pain
Gregorini, 2010, Brazil [58]	Pain, respiratory function	59.9 (10.3)	25: (13/12)	72%	TENS Sham TENS	-	-	Elective cardiac surgery	Third post-op day	VAS	Reduces pain
Jahangirifard, 2018, Iran [61]	Pain, respiratory function, narcotics use, drain secretions, ICU LoS, N requests for chest radiographs	58.4 (8.1), 60.1 (6.6)	100: (50/50)	50%/50%	TENS Sham TENS	-	-	Elective coronary artery bypass	Post-op 30 min every 4 h	VAS 0–10	Reduces pain, better pulmonary function
Lima, 2011, Brazil [39]	Pain, MIP, MEP	54.2 55.1	20 (10/10)	50%	TENS No TENS	CAD	-	CABG	30 min, 3 times a day, 3 h each	VAS 0–10	Reduces pain; increase in respiratory muscle strength
Navarathnam, 1984, Australia [65]	Pulmonary function, analgesic use, atelectasis, pain	56.4 (39–67) 52.2 (17–69)	31 (14/17)	86%/77%	TENS Sham TENS	CAD, valve disease	-	CABG, AV replacement, MV replacement	-	Digital scoring system (1–5)	May be of benefit in post-op pain relief
Sezen, 2017, Turkey [50]	Post-op pain, complications	55.13 (14.63), 58.86 (11.82)	87: (43/44)	74%/68%	TENS Sham TENS	-	-	Thoracotomy	8 h post-op	VAS 0–10	No effect on hospital stay, complications; safe pain management
Solak, 2007, Turkey [69]	Pain, pulmonary function	47.3 (11.7) 53.72 (12.6)	40 (20/20)	70%/90%	TENS PCA	-	-	Posterolateral thoracotomy	4 h post-op	VAS, Prince Henry score	Better pain relief than PCA
Stubbing, 1988, UK [70]	Analgesic use, time to oral analgesia, antiemetic use, LOS, pulmonary function	54 (17.8) 53 (15.7)	40 (20/20)	65%/75%	TENS + IM papaveretum IM papaveretum alone	-	-	Thoracotomy	For 48 h post-op	0–4	Lower PONV, no effect on analgesia use, peak expiratory flow rate
Kara, 2011, Turkey [48]	Pain, function, depression, side effects	45.62 (10.59) 47.60 (13.75)	54 (25/29)	40%/55%	TENS + PCA PCA only	-	-	Open lumbar discectomy	Twice for 30–40 min, 3–4 h interval	Horizontal 100 mm VAS	Reduces side effects, analgesic use, activity-related pain
McCallum, 1988, UK [64]	Morphine use	44.6 (9.1), 45.7 (11.7)	20: (10/10)	50%/20%	TENS Sham TENS	-	-	Lumbar laminectomy	12 h prior to surgery	-	No effect on pain
Parseliunas, 2020, Lithuania [49]	Pain, analgesics use	61.77 (10.84), 61.08 (12.51)	80: (40/40)	100%	TENS Sham TENS	Unilateral inguinal hernia	-	Open inguinal hernia repair	Post-op	100 mm VAS	Reduces post-op pain
Smedley, 1988, UK [51]	Pain, analgesic use, peak expiratory flow	57 (21–83) 55 (24–78)	62 (34/28)	100%	TENS Sham TENS	Inguinal hernia	-	Inguinal hernia repair	48 h post-op	LAS	No differences
Chen, 2021, China [55]	Pain, pain attacks, N/amount analgesic drugs, changes in gene expression	73%: 20–35	70 (35/35)	0%	TENS + analgesic drugs Analgesic drugs only	-	-	Elective C-section	24 h post-op, 30 min each	10 cm VAS	Reduces pain, N pain attacks, analgesic use, and expression of PNMT gene
Kurata, 2022, USA [47]	Opioid use, pain, patient satisfaction, LOS, adverse events	31 (6) 32 (6) 31 (6)	180 (60/60/60) ITT	0%	TENS Sham TENS No TENS	Obstetric	Prior c-section, other uterine incision	C-section	30 min post-op, until discharge, PCA	0–10 Likert scale	No effect on opioid use, pain, LOS
da Silva, 2015, Brazil [35]	Pain, analgesic use, adverse effects, quality of pain, treatment success, patient satisfaction	25 27	42 (21/21)	100%	TENS Sham TENS	-	-	Liposuction	30 min post-op	-	Effective in adjunction to analgesics for pain
Erden, 2022, Turkey [45]	Pain, patient satisfaction	57.1 (10.88) 56.9 (10.2)	80 (40/40)	0%	TENS No TENS	Breast cancer	Chronic diseases	Mastectomy	2 times for 20 min	NRS 0–10	Useful analgesic method
Ilfeld, 2021, USA [34]	Opioid use, pain, QoL	56.8 (15.8) 55.4 (15.9)	65 (31/34)	52%/50%	TENS Sham TENS	ACL injury, rotator cuff injury, hallux valgus, ankle arthrodesis, arthroplasty	-	Major foot/ankle surgery, anterior cruciate ligament reconstruction, rotator cuff repair	Up to 14 d post-op, daily	Average daily NRS 0–10	Reduces pain and opioid use, no systemic side effects
Mahure, 2017, USA [59]	Anesthetic use, pain	60.5 (11.1) 56.4 (12.2)	37 (21/16)	53%/44%	TENS Sham TENS	-	-	Arthroscopic rotator cuff repair	-	VAS	Less pain, opioid use
Wang, 2014, China [71]	Intraoperative remifentanil use, side effects	43.1 (15.0) 39.9 (15.7	60 (30/30)	53%/63%	TEAS Sham TEAS	-	-	Sinusotomy	30 min before anesthesia	-	Less incidence of side-effects

**Table 2:** Risk of Bias table. “+” low risk of bias; “?” some concerns; “-” high risk of bias.

First Author, Year	1	2	3	4	5	6
Asgari 2018 [37]	+	?	+	+	+	?
Beckwee 2017 [41]	?	+	+	+	+	?
Benedetti 1997 [66]	?	?	+	+	+	?
Bjersa 2014 [62]	+	?	+	+	+	?
Bjersa 2015 [38]	+	?	+	+	+	?
Chen 2021 [55]	?	?	+	+	+	?
Chiu 1999 [42]	+	?	+	+	+	?
Cuschieri 1985 [63]	?	+	+	+	+	?
da Silva 2015 [35]	+	?	?	+	+	?
Elboim-Gabyzon 2019 [43]	+	+	+	+	+	+
Engen 2015 [44]	?	?	+	+	+	?
Erden 2022 [45]	+	+	-	-	+	+
Erden 2016 [36]	+	?	+	+	+	?
Erdogan 2005 [46]	?	+	+	+	+	?
Ferreira 2011 [40]	?	?	+	+	+	?
Fiorelli 2011 [2]	+	+	+	+	+	+
Forogh 2017 [56]	+	+	+	+	+	+
Galli 2015 [57]	+	+	+	+	+	+
Gregorini 2010 [58]	+	?	+	+	+	?
Hamza 1999 [67]	+	?	?	+	+	?
Ilfeld 2021 [34]	+	+	+	+	+	+
Jahangirifard 2018 [61]	?	+	+	+	+	?
Kara 2011 [48]	+	?	+	+	+	?
Kurata 2022 [47]	+	+	+	+	+	+
Laitinen 1991 [68]	?	?	+	+	+	?
Lima 2011 [39]	-	?	?	?	?	-
Mahure 2017 [59]	+	?	+	?	+	?
McCallum 1988 [64]	?	+	+	+	+	?
Navarathnam 1984 [65]	?	?	+	+	+	?
Parseliunas 2020 [49]	+	+	+	+	+	+
Rakel 2003 [60]	?	?	+	+	+	?
Sezen 2017 [50]	?	?	+	+	+	?
Silva 2012 [13]	+	?	+	+	+	?
Smedley 1988 [51]	+	?	+	+	+	?
Solak 2007 [69]	?	?	+	+	+	?
Stubbing 1988 [70]	?	?	+	+	+	?
Wang 2014	+	+	+	+	+	+
Yu 2020 [52]	+	+	+	+	+	+
Zhang 2017 [53]	+	?	+	+	+	?
Zhang 2018 [54]	?	?	+	+	+	?

1. Risk of bias arising from the randomization process. 2. Risk of bias arising from deviations from the intended interventions. 3. Risk of bias arising from missing outcome data. 4. Risk of bias arising from the measurement of the outcome. 5. Risk of bias arising from the selection of the reported results. 6. Overall risk of bias.

**Table 3:** Summary of findings. ⨁⨁⨁⨁ high quality of evidence, ⨁⨁⨁⊖ moderate quality of evidence, ⨁⨁⊖⊖ low quality of evidence, ⨁⊖⊖⊖ very low quality of evidence. Population: Patients undergoing various surgeries. Settings: In-hospital. Intervention: Use of TENS. Comparison: No TENS [2,13,34,35,36,37,38,39,40,41,42,43,44,45,46,47,48,49,50,51,52,53,54,55,56,57,58,59,60,61,62,63,64,65,66,67,68,69,70,71].

Outcomes	Risk Ratio (95% CI)	(Standardized) Mean Difference [95% CI]	N of Participants (Studies)	Certainty of the Evidence (GRADE)
Pain at rest, 24 h (0–10)	-	−0.69 [−1.33, −0.06]	1136 (18)	⨁⨁⊖⊖ Very low ^a^
Morphine requirements, 24 h (mg)	-	−15.64 [−26.69, −4.58]	361 (7)	⨁⊖⊖⊖ Very low ^b^
Postoperative nausea	0.46 [0.21, 1.04]	-	265 (5)	⨁⨁⊖⊖ Low ^c^
Pruritus	0.44 [0.26, 0.76]	-	226 (3)	⨁⨁⨁⊖ Moderate ^d^
Hospital stay duration (days)	-	−1.16 [−2.35, 0.02]	514 (7)	⨁⨁⊖⊖ Low ^e^

^a^ For three studies, the randomization method was unclear. Four studies were not blinded, and for two, blinding was unclear. There was considerable heterogeneity, wide variance of point estimates, and some confidence intervals did not overlap. ^b^ Four studies did not specify randomization procedures. One study was not blinded, and one study did not mention blinding. There was considerable heterogeneity, wide variance of point estimates, and some confidence intervals did not overlap. The confidence interval of the pooled effect crossed the no-difference line. ^c^ For two studies, the randomization method was unclear. Two studies were not blinded, and for two studies, blinding was unclear. There was moderate heterogeneity. The overall risk ratio was lower than 0.5; therefore, the outcome was upgraded. ^d^ For one study, the randomization method was unclear, for another, blinding was unclear. The overall risk ratio was lower than 0.5. ^e^ Four studies did not properly describe the randomization process. One study was not blinded, and two did not mention blinding. There was considerable heterogeneity and a wide variance of point estimates.

**Table 4:** The evidence profile (the quality of evidence evaluation and the summary of findings for each of the studied outcomes).

	**Pain at Rest, 24 h**	**Morphine Requirements, 24 h (mg)**	**Postoperative Nausea and Vomiting**	**Pruritus**	**Hospital Stay Duration (Days)**
**Risk of Bias**	**Very Serious**	**Very Serious**	**Very Serious**	**Serious**	**Very Serious**
Lack of allocation concealment	Some concerns	Some concerns	Some concerns	No	Some concerns
Lack of blinding	Some concerns	Some concerns	Some concerns	No	Some concerns
Incomplete accounting of patients and outcome events	Some concerns	Some concerns	Some concerns	Some concerns	Some concerns
Selective outcome reporting	No	No	No	No	No
Other limitations	No	No	No	No	No
**Inconsistency**	**Very serious**	**Very serious**	**Serious**	**Not serious**	**Serious**
I^2^ (unexplained heterogeneity of results)	Considerable	Considerable	Moderate	None	Considerable
Wide variance of point estimates	No	Yes	Yes	No	Yes
Confidence intervals (CIs) do not overlap	Yes	Yes	No	No	No
**Indirectness**	**Not serious**	**Not serious**	**Not serious**	**Serious**	**Not serious**
Differences in population	No	No	No	No	No
Differences in interventions	No	No	No	No	No
Differences in outcome measures	No	No	No	Yes	No
Indirect comparisons	No	No	No	No	No
**Imprecision**	**Not serious**	**Serious**	**Not serious**	**Not serious**	**Not serious**
Few patients	Not serious	Not serious	Not serious	Not serious	Not serious
Wide confidence interval (CI)	Not serious	Serious	Not serious	Not serious	Not serious
**Upgrading**	**None**	**None**	**None**	**RR < 0.5**	**None**
RR > 2 or RR < 0.5 RR > 5 or RR < 0.2	No	No	No	RR < 0.5	No
Dose-response gradient	No	No	No	No	No
Effect of plausible residual confounding	No	No	No	No	No

## Data Availability

The data can be requested from the corresponding author.
